# Assessment of Astaxanthin Accumulation in Hepatocytes of Atlantic Salmon Fed Different Diets Using NMR Spectroscopy

**DOI:** 10.3390/molecules25081769

**Published:** 2020-04-12

**Authors:** Elena Shumilina, Alessandra Ciampa, Trine Ytrestøyl, Alexander Dikiy

**Affiliations:** 1 Department of Biotechnology and Food Science, Norwegian University of Science and Technology (NTNU), 7491 Trondheim, Norway; alessandraciampa@libero.it (A.C.); alex.dikiy@ntnu.no (A.D.); 2 NOFIMA, 6600 Sunndalsøra, Norway; trine.ytrestoyl@nofima.no

**Keywords:** carotenoid, astaxanthin, Atlantic salmon, fish diet, fish feed, plant proteins, DHA, hepatocytes, NMR

## Abstract

This study aimed to assess the astaxanthin (Ax) accumulation in hepatocytes isolated from farmed Atlantic salmon fed different diets (rich marine, poor, poor with marine phospholipids (MPL) and poor with docosahexaenoic acid (DHA)). Nuclear magnetic resonance (NMR) spectroscopy was used for the Ax detection and quantification. The use of the ^13^C-enriched Ax allowed the assessment of short-time Ax metabolism. The substitution of fish oil and meal in fish feed on plant analogs and the addition of MPL caused further catabolism and decrease of Ax accumulation in hepatocytes from 17 to about 6 mg/kg or to almost zero in the case of DHA addition. Signals assignment of the native and ^13^C-enriched astaxanthin in acetone were performed using 1D and 2D NMR spectra.

## 1. Introduction

Salmonid are able to deposit astaxanthin (Ax) and canthaxanthin in their white muscle [1]. These carotenoids refer to the fish muscle the typical pink color. Astaxanthin is the most abundant carotenoid in the flesh of wild Atlantic salmon [2,3]. Fish should obtain Ax from external sources to acquire the appropriate coloration [1,4,5]. Unfortunately, only 4–15% of the dietary Ax is used for flesh pigmentation [4,6,7]. Ax uptake and deposition depends on different factors. The effect of different types of feed pigments, sources of fish oil, additives and fish life stage on the pigment deposition in flesh of farmed Atlantic salmon have been the objects of scientific studies and are well reviewed by Lim et al. [5].

Poor deposition of astaxanthin in skeletal muscle is a serious concern for the aquaculture industry because flesh coloration is essential for the consumers’ perception [8]. A satisfactory coloration of salmon flesh might be achieved when the concentration of Ax in salmon muscles exceeds 6–7 mg/kg [1]. To achieve this level, aquaculture should increase the Ax content in commercial feeds. The Panel on Additives and Products or Substances used in Animal Feed (FEEDAP) reports a maximum dietary limit of 100 mg of astaxanthin for one kg of fish feed [9].

The biological mechanisms of Ax uptake, metabolism and deposition were subjects of different studies reviewed by Lim et al. [5]. Astaxanthin might be catabolized at different stages, including digestion and intestinal absorption, blood transportation and tissue uptake, oxidation and conversion. Most carotenoids are metabolized in the liver [10,11]. The complexity of the problem implies the demand on the further development of the analytical methods for the assessment of the astaxanthin uptake and turnover in each catabolic stage. NMR spectroscopy might provide detailed information on Ax metabolism in different salmon’s tissues. This method facilitates the detection and quantification of Ax and its derivatives: idoxanthin, β-carotene, retinol and others [12,13].

The present study is a further development of NMR methods for the determination of carotenoid metabolism in salmonids. Here, 1D and 2D NMR spectroscopy was used to quantify ^13^C-enriched astaxanthin accumulated by hepatocytes, since liver metabolism has a major responsibility for the metabolic loss of astaxanthin [14]. Cells were isolated from farmed Atlantic salmon fed different diets. Our study assesses how partial substitution of fish meal and oil on plant analogs influences astaxanthin accumulation in hepatocytes.

## 2. Results and Discussion

Two different types of astaxanthin were used in the current study. First, natural astaxanthin (Ax) was part of the salmon diet (Table 1, Carophyll pink containing 10% of astaxanthin). Second, enriched at 7,7′-8,8′ position, ^13^C-astaxanthin was supplied to the isolated hepatocytes (13C-Ax). The Ax enrichment allowed us to assess 1) the presence of the natural astaxanthin in the cells (due to the long-term astaxanthin accumulation in the fish’s liver) and 2) the amount of enriched astaxanthin from the short-term carotenoid assimilation by incubated cells. In order to detect and quantify both types of astaxanthin, an assignment of NMR resonances of natural and enriched astaxanthin was carried out as described below.

### 2.1. Assignment of Natural and 7,7′,8,8′-^13^C-Enriched Astaxanthin

**Figure 1:**
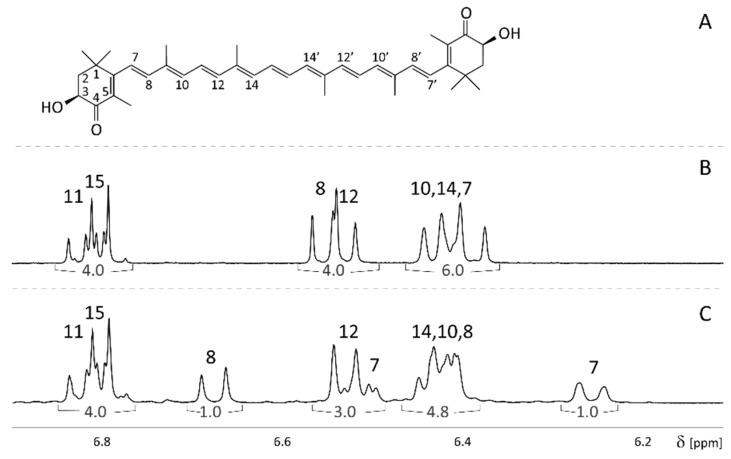
The structure of astaxanthin (**A**) and ^1^H NMR spectra of the astaxanthin standards in acetone at 300 K (600 MHz). (**B**) Natural astaxanthin (Ax); (**C**) 7,7′, 8,8′-^13^C-enriched astaxanthin (13C-Ax). The numbers over the spectra show the resonance assignment (symmetrical hydrogens are shown with the same number). The numbers in buckets below the spectra show the relative integral values. δ – chemical shift in parts per million (ppm).

The chemical shifts of the resonances of enriched 7,7′,8,8′-^13^C astaxanthin (13C-Ax) are rather different from its natural analog (Ax). While the NMR spectrum of natural astaxanthin in chloroform was already discussed by Englert [12], the 13C-Ax resonances in acetone have not yet been assigned. Here, we are reporting the resonances assignment of Ax and 13C-Ax in acetone (Figure 1). Symmetrical hydrogens are shown with the same number (for example, hydrogens 7 and 7′ will be referred to as hydrogen 7, Figure 1).

The chemical shift and J-coupling of the hydrogens 11, 15, 12, 10 and 14 are similar in Ax and 13C-Ax. However, the ^13^C enrichment at the position 7 and 8 causes a shift of the H(7) and H(8) resonances. The satellite signals of the H(7) and H(8) caused by the ^1^H-^13^C coupling are not observed in the ^1^H spectra because the natural abundance of ^13^C is only approximately 1%. Therefore, the 2-spin system of H(7) and H(8) can be identified as an AB-type spectrum with J_7,8_ = 15.5–16.8 Hz in the ^1^H spectrum of natural astaxanthin. Because astaxanthin has a β-end group, the H(7) doublet can be distinguished from the H(8) doublet by its larger line width [12]. The spectrum of natural astaxanthin (Ax) has two doublets at 6.55 and 6.39 ppm with J_7,8_ = 16.1 and 16.3Hz, respectively. The signal at 6.39 ppm has a larger line width and a lower peak height and was assigned as H(7).

**Figure 2:**
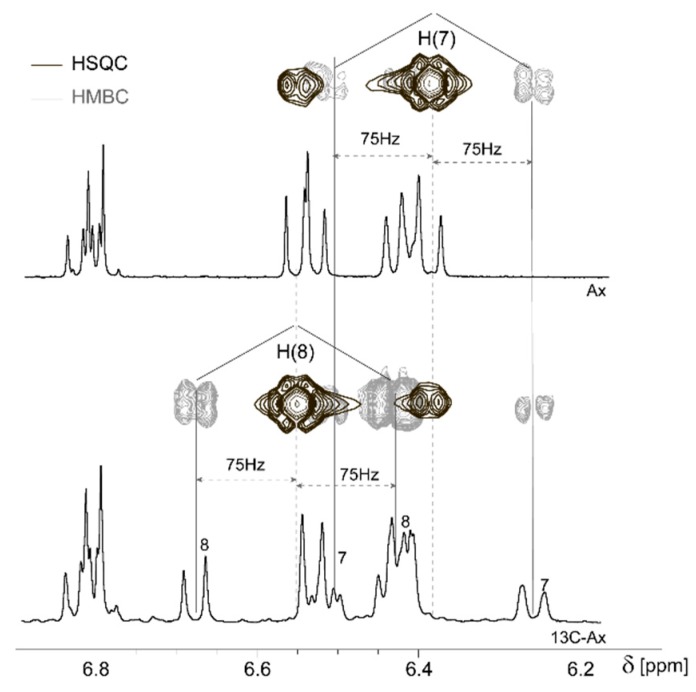
One-dimensional (1D) ^1^H and two-dimensional (2D) ^1^H-^13^C HSQC (black) and ^1^H-^13^C HMBC (gray) spectra of ^13^C-enriched astaxanthin (13C-Ax) at 300 K. Ax-1D ^1^H NMR spectrum of natural astaxanthin. δ—chemical shift in ppm.

The substitution of ^12^C(7) and ^12^C(8) carbons by ^13^C allows the detection of the ^1^H-^13^C coupling. This phenomenon is observed in the ^1^H spectrum of 13C-Ax as two satellite signals on the sides of the main uncoupled signal that is not seen anymore. Theoretically, the one bond ^1^H-^13^C coupling constants for the compounds with a sp^2^-hybridization could be expected between 115 and 250 Hz [15,16]. The 2D ^1^H-^13^C HSQC and HMBC spectra help to determine the coupling constant. Figure 2 shows that the ^1^H-^13^C HSQC spectrum of 13C-Ax contains a cross peak at 6.55/142 and 6.39/124 ppm (Figure 2, black line). The used pulse program (*hsqcetgpprsisp2.2*) reveals that the chemical shift of H(7) and H(8) in 13C-Ax is similar to that of the natural compound. On the other hand, the spectrum of the ^1^H-^13^C HMBC experiment (*hmbcgplpndprqf* pulse program, Figure 2, gray line) contains two pairs of satellite cross peaks on both sides from the HSQC cross peaks (6.26/124 and 6.52/124; 6.68/142 and 6.43/142 ppm, Figure 2). These HMBC cross peaks have the same chemical shift as the resonances in the lower 1D ^1^H spectrum of 13C-Ax (Figure 2). Because the satellite resonances of H(7) and H(8) are located at a distance of 150 Hz, it can be concluded that the coupling constants ^1^J(C_7_,H_7_) = ^1^J(C_8_,H_8_) are 150 Hz. This value is in agreement with the theoretical value reported above. Despite the satellite signals usually maintaining the coupling pattern of the main peak, the presence of other ^13^C and ^12^C neighboring atoms breaks the symmetry of the multiplet structure of the H(7) satellites at 6.51 ppm (Figure 2, lower 1D ^1^H NMR spectrum). The *hsqcetgpprsisp2.2* pulse program removes the satellite cross peaks. Therefore, the ^1^H-^13^C HSQC spectra of the hepatocytes extracts and natural carotenoids standards could be compared in order to assign the 13C-Ax derivatives.

The differences in the chemical shifts of the H(7) and H(8) protons in natural and enriched astaxanthin allowed us to unambiguously distinguish them. Enriched astaxanthin (13C-Ax) has doublets at 6.68 and 6.26 ppm (J = 16 and 16.7 Hz, respectively), while natural astaxanthin can be identified by the group of two doublets at 6.55 ppm.

### 2.2. Astaxanthin Detection and Quantification in Hepatocytes

**Figure 3:**
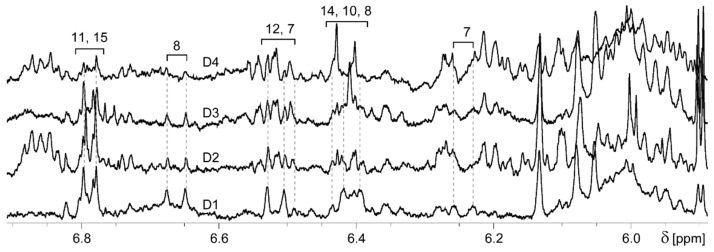
Comparison of the 6.9–5.5 ppm region of ^1^H NMR spectra of the acetone extracts of salmon hepatocytes incubated with ^13^C-enriched astaxanthin for 48 h. D1, D2, D3 and D4–samples from diet 1, 2, 3 and 4, respectively. The numbers over the spectra show the 13C-Ax resonance assignment. δ—chemical shift in ppm.

The astaxanthin accumulation in hepatocytes isolated from the farmed Atlantic salmon fed different diets (rich marine (D1), poor (D2), poor with marine phospholipids (D3) and poor with docosahexaenoic acid (DHA) (D4)) was assessed after the resonance assignment. Natural astaxanthin (Ax) was not detected in any of the hepatocytes. However, the ^1^H NMR spectra of hepatocytes acetone extracts contain resonances that belong to various carotenoids (Figure 3).

**Figure 4:**
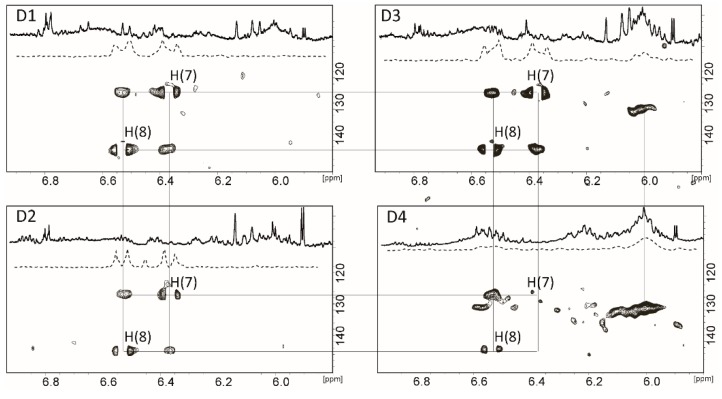
^1^H and ^1^H-^13^C HSQC spectra of acetone extracts of salmon hepatocytes at 300 K. The dashed spectra represent the 1D projections of ^1^H-^13^C HSQC spectra.

The 13C-Ax accumulation in hepatocytes was confirmed by 2D ^1^H-^13^C HSQC NMR spectra. The intensive cross peaks of ^13^C-(H7) and 13C-(H8) are well visible in the ^1^H-^13^C HSQC spectra (Figure 4). From the 1D projection of ^1^H-^13^C HSQC (Figure 4, dashed lines), it can be seen that the intensity of the cross peaks of the enriched carbons is significantly higher than the intensity of the other cross peaks from the same molecule. The characteristic system of ^1^H(7)-^13^C(7) and ^1^H(8)-^13^C(8) cross peaks is well defined in the spectra of the diets 1–3. Diet 4 only has a trace amount of 13C-Ax.

Furthermore, diets 3 and 4 contained a product of the 13C-Ax metabolism (peak at 6.0–6.1/129.8 ppm, Figure 4). The intensity of the cross peak suggests that it belong to the ^13^C-enriched carotenoid, instead of the natural astaxanthin. The ^1^H-^13^C HSQC spectrum of D4 shows that the main change occurs with the H(7) signal at 6.37/124.5 ppm: its correspondent cross peak is rather weak in D4. The possible high field shift of the H(7) proton from 6.37 to 6.01 ppm can be due to the formation of an epoxy group between the C(5) and C(6) atoms of the end group [12]. Therefore, the enrichment of the salmon feed with the phospholipids or DHA might cause astaxanthin oxidation and formation of the peroxide in the isolated hepatocytes. This can lead to the further deviations in the astaxanthin transport or deposition in other tissues.

The doublet at 6.68 ppm was used to quantify the 13C-Ax in NMR spectra of acetone extracts of isolated hepatocytes. The D1 hepatocytes (rich marine diet) contain a higher amount of astaxanthin (17 mg/kg) compared to the poor marine diet (D2) and the diet with marine phospholipids (MPL) (D3) (5.3–6.3 mg/kg) cells. Astaxanthin was found in trace amounts in the hepatocytes isolated from fish fed D4. NMR spectra of the D2-D4 hepatocytes were found to contain resonances that could belong to the polyenic chain of other carotenoids (Figure 3 and Figure 4). Therefore, the lower amount of accumulated 13C-Ax in diets D2-D4 is most probably due to the further metabolism of astaxanthin and not due to a decreased uptake. As it was discussed in the Introduction, a satisfactory coloration of salmon flesh might be achieved when the concentration of Ax in salmon muscles exceeds 6–7 mg/kg. Our study shows that, the substitution of fish oil and meal in fish feed on plant analogs and the addition of MPL or DHA caused the decrease of Ax accumulation in hepatocytes that may hinder Ax supply to other organs.

## 3. Materials and Methods

### 3.1. Experimental Design

Four groups of Atlantic salmon were fed four different diets. The reference group obtained a rich marine diet based on fish meal and oil. The content of fish meal and oil was reduced, and plant proteins and plant oil were added to the feed of other groups. MPL and DHA were added to diet 3 and diet 4, respectively. All feed contained 50 mg/kg of astaxanthin.

Hepatocytes were isolated from six fish from each group after 72 days of fish feeding. Isolated cells were incubated for 48 h in the medium containing ^13^C-enriched astaxanthin.

Harvested hepatocytes were collected and sent to NMR analysis. Astaxanthin was extracted from all hepatocytes with acetone. Then, 1D and 2D NMR spectra were acquired to determine astaxanthin accumulation and catabolism.

### 3.2. Salmon Feeding

All feeding experiments were carried out at the experimental station of Nofima, Sunndalsøra, Norway. Before the experiment started, the fish were fed a commercial diet without carotenoid supplements (Skretting, Stavanger, Norway). Upon the start of the experiment, triplicate groups of size-graded Atlantic salmon (*Salmo salar*) with an initial average weight of 197 g were fed four experimental diets for a period of 72 days (Table 1). The reference group was fed a rich diet based on marine ingredients: fish meal and fish oil (D1). The second group (D2) was fed a poor marine diet containing soy protein concentrate and wheat gluten as the main protein sources and rapeseed oil as the major oil source (D2). Diet 3 (D3) was identical to Diet 2 except that it replaced some of the fish oil with a MPL product made from fish meal. Diet 4 (D4) consisted of D2 where fish oil was replaced with a DHA concentrate. All experimental diets were fortified with 50 mg/kg of natural astaxanthin (Carophyll pink). Fish were maintained indoors in 1 m^2^ fiberglass tanks (600 L) supplied with saltwater, with an average temperature of 12.1 °C. The tanks were supported with continuous illumination. The fish were fed in excess to ensure the highest possible feed intake during each meal. After 72 days, hepatocytes were isolated from two fish per tank (six per treatment) as described in Section 3.3.

### 3.3. Hepatocytes Isolation

Hepatocytes were isolated from six salmons from each dietary group. Fish were anesthetized in Metacain (MS-222), the abdominal cavity was exposed, and the vena porta was cannulated. The liver was perfused in a two-step collagenase procedure according to the method described in Kjær et al. [17]. The digested liver tissue was filtrated through a 100 μM nylon filter to separate the hepatocytes from tissue debris. The cells were then washed three times in L-15 medium (Sigma-Aldrich, St. Louis, MO, USA) and centrifuged for 2 min at 50× *g* between each wash. Finally, the cells were resuspended in L-15 GlutaMAX (Invitrogen, Carlsbad, CA, USA) growth medium containing 10% fetal calf serum (FCS) (PAA Laboratories GmbH, Pasching, Austria), 1% bicarbonate (Sigma-Aldrich, St. Louis, MO, USA), 5 mM Hepes (Sigma-Aldrich, St. Louis, MO, USA) and 1% PenStrep (Sigma-Aldrich, St. Louis, MO, USA). Cell viability was assessed by Trypan blue staining, 0.4% (Sigma-Aldrich, St. Louis, MO, USA). Approximately 15 million hepatocytes were seeded out in 25 cm^2^ cell flasks coated with laminin (Sigma-Aldrich, St. Louis, MO, USA). Cells were cultured at 13 °C overnight and then the experimental growth media were added.

Isolated hepatocytes from all groups were incubated with the experimental growth medium L-15 GlutaMAX containing 2% FCS, 1% bicarbonate, 5 mM HEPES, and 1% PenStrep, in addition to 0.9 µg/mL 7,7′,8,8′-^13^C-astaxanthin (Buchem B.V., Apeldoorn, Netherlands). All cells were incubated for 48 h at 13 °C. The cells were washed once in 1% BSA in phosphate buffer saline (PBS) (Sigma-Aldrich, St. Louis, MO, USA) and twice in PBS before harvesting in PBS. The harvested cells were centrifuged for 2 min at 3000× *g*, the supernatant was removed, and the cell pellets were stored at −80 °C until analysis.

### 3.4. Astaxanthin Extraction from Hepatocytes

Consecutive hexane and acetone extractions of hepatocytes (7–30 mg) were used to separate non-polar metabolites from more polar carotenoids. For that, 200 µL of hexane was added to cells. The cells were homogenized with a ball mill homogenizer (Retch Mixer Mill MM 400, (Retsch, Haan, Germany) 2 min; 30 v/s; 4 °C; three glass balls). The homogenates were centrifuged for 10 min at 20,000× *g*, 4 °C, and the pellet was used for the other five hexane extractions. The supernatants from all the extractions were collected together and stored at 4 °C. Astaxanthin was not found in the hexane fraction. Therefore, the pellet was extracted again using 200 µL acetone, which was added to the pellet. The extraction was carried out by Retch (2 min; 30 v/s; 4 °C; three glass balls). The homogenate was centrifuged for 10 min at 20,000× *g*, 4 °C, and the pellet was used for the other five acetone extractions. Supernatants from all acetone extractions were collected separately from the hexane supernatants and stored at 4 °C. The organic solvents (hexane and acetone) and trace amount of water were removed from both extracts, and the resulting solid residues were stored at −80 °C.

### 3.5. NMR Sample Preparation

A total of 200 µL of the deuterated acetone (acetone-d6, 99.9 atom % D, with 0.03% *v/v* of tetramethylsilane (TMS) from Acros Organics, Switzerland) was added to the lyophilized samples of the acetone extracts of hepatocytes right before the NMR experiments. Samples were mixed and centrifuged (10 min at 20,000× *g* at 8 °C) and then transferred into 3 mm NMR tubes. Pure deuterated acetone containing 0.03% (*v*/*v*) TMS was used as an external standard for the carotenoid’s quantification.

### 3.6. NMR Data Acquisition and Processing

1D ^1^H NMR spectra of acetone extracts and TMS standard in acetone were acquired at 300 K with a Bruker Avance 600 MHz spectrometer equipped with a 5 mm z-gradient TXI (H/C/N) cryoprobe. The water pre-saturation NMR experiments were acquired with the Bruker pulse sequence *noesygppr1d* using the following settings: relaxation delay (d1) = 2 s; SW = 20 ppm; NS = 4 K; RG = 64. The Topspin 3.5pl7 software (Bruker, Germany) was used for the calibration of spectra, baseline correction and resonance integration. The signal of the TMS at 0 ppm was used for the calibration of all spectra. The baseline was corrected manually. An integral value used for the carotenoid quantification was an average value of three integrations of the same signal.

2D ^1^H-^1^H TOCSY, ^1^H-^13^C HSQC, ^1^H-^13^C HMBC and J-RES NMR spectra of the Ax standard for resonance assignment were acquired using the standard Bruker pulse sequences (mlevesgpph, hsqcetgpprsisp2.2, hmbcgplpndprqf and jresgpprqf, respectively).

## 4. Conclusions

In this study, 1D and 2D NMR spectroscopy was used to assess the astaxanthin accumulation by the isolated from Atlantic salmon hepatocytes cells. Natural and ^13^C-enriched astaxanthin resonance assignment in acetone was carried out in ^1^H and ^1^H-^13^C HSQC and ^1^H-^13^C HMBC spectra. The addition of the 7,7′,8,8′-^13^C astaxanthin to the cell’s growth medium enabled us to distinguish the long-term accumulation of the astaxanthin and the short-term carotenoid accumulation and catabolism in isolated hepatocytes. Any natural ^12^C astaxanthin was detected in the studied cells. At the same time, the hepatocytes previously exposed to the rich marine diet were able to accumulate 17 mg/kg of the ^13^C-enriched astaxanthin from the medium containing 0.9 mg/L of 13C-Ax. Differences in diet composition significantly influenced the short-term astaxanthin metabolism in hepatocytes. For example, the addition of DHA to the poor diet (D4) caused a decrease in astaxanthin concentration to almost zero. The proposed approach might be used for the further assessment of the astaxanthin uptake and metabolism in different isolated cells and salmon tissues.

## Figures and Tables

**Table 1:** Diet composition. D1—rich marine diet; D2—poor marine diet; D3—poor marine diet fortified with marine phospholipid (MPL); D4—poor marine diet fortified with docosahexaenoic acid (DHA).

Ingredient (%)	Product Name, Supplier	D1	D2	D3	D4
Fishmeal	Norse-LT, Vedde AS, Langevåg, Norway	58.70	7.50	7.50	7.50
Fish oil	NorSalmOil, Pelagia, Egersund, Norway	22.00	6.50	3.30	0.50
Wheat	Norgesmøllene AS, Bergen, Norway	13.50	10.00	10.00	10.00
Wheat gluten	Amytex 100, Tereos Syral, Aalst, Belgium	-	22.45	22.45	22.45
Soy protein concentrate	Alpha-Soy Pro 650, Agrokorn, Videbaek, Denmark	-	26.00	26.00	26.00
Rapeseed oil	Crude rapeseed oil, Emmelev, Otterup, Denmark	-	19.40	19.40	19.40
Mineralmix	Nofima Mineralpremix, Vilomix, Hønefoss, Norway	0.59	0.59	0.59	0.59
Vitaminmix	Nofima Vitmainpremix, Vilomix, Hønefoss, Norway	2.00	2.00	2.00	2.00
MSP (26% P)		2.50	2.50	2.50	2.50
Carophyll pink (10% Astaxanthin)	Carophyll pink, Vilomix, Hønefoss, Norway	0.05	0.05	0.05	0.05
Yttrium oxide	Diyttrium trioxide 99.9%, VWR, Oslo, Norway	0.01	0.01	0.01	0.01
Betafine	Betafine, delivered by Vilomix, Hønefoss, Norway	0.50	0.50	0.50	0.50
DL-Methionine	DL-Methionine, delivered by Vilomix, Hønefoss, Norway	0.20	0.80	0.80	0.80
L-Lysine	L-lysine, delivered by Vilomix, Hønefoss, Norway	-	1.70	1.70	1.70
Threonine	L-Threonine, delivered by Vilomix, Hønefoss, Norway	-	0.10	0.10	0.10
Marine phospholipids	Marine phospholipids, TripleNine, Esbjerg, Denmark	-	-	3.20	-
DHA	Incromega DHA 500TG, Croda Nordica AB, Limhamn, Sweeden	-	-	-	6.00
Mineralmix	Nofima Mineralpremix, Vilomix, Hønefoss, Norway	0.59	0.59	0.59	0.59
